# Targeted De-Methylation of the FOXP3-TSDR Is Sufficient to Induce Physiological FOXP3 Expression but Not a Functional Treg Phenotype

**DOI:** 10.3389/fimmu.2020.609891

**Published:** 2021-01-07

**Authors:** Christopher Kressler, Gilles Gasparoni, Karl Nordström, Dania Hamo, Abdulrahman Salhab, Christoforos Dimitropoulos, Sascha Tierling, Petra Reinke, Hans-Dieter Volk, Jörn Walter, Alf Hamann, Julia K. Polansky

**Affiliations:** ^1^ Berlin Institute of Health Center for Regenerative Therapies (BCRT), Charité - Universitätsmedizin Berlin, Berlin, Germany; ^2^ Immuno-Epigenetics, German Rheumatism Research Centre (DRFZ), Berlin, Germany; ^3^ Genetics/Epigenetics, Saarland University, Saarbrücken, Germany; ^4^ Berlin Center for Advanced Therapies (BeCAT), Charité - Universitätsmedizin Berlin, Berlin, Germany

**Keywords:** T cell differentiation, regulatory T cells, epigenetic editing, adoptive T cell therapies, gene regulation, CRISPR-Cas9-based tool

## Abstract

CD4+ regulatory T cells (Tregs) are key mediators of immunological tolerance and promising effector cells for immuno-suppressive adoptive cellular therapy to fight autoimmunity and chronic inflammation. Their functional stability is critical for their clinical utility and has been correlated to the demethylated state of the TSDR/CNS2 enhancer element in the Treg lineage transcription factor FOXP3. However, proof for a causal contribution of the TSDR de-methylation to FOXP3 stability and Treg induction is so far lacking. We here established a powerful transient-transfection CRISPR-Cas9-based epigenetic editing method for the selective de-methylation of the TSDR within the endogenous chromatin environment of a living cell. The induced de-methylated state was stable over weeks in clonal T cell proliferation cultures even after expression of the editing complex had ceased. Epigenetic editing of the TSDR resulted in FOXP3 expression, even in its physiological isoform distribution, proving a causal role for the de-methylated TSDR in FOXP3 regulation. However, successful FOXP3 induction was not associated with a switch towards a functional Treg phenotype, in contrast to what has been reported from FOXP3 overexpression approaches. Thus, TSDR de-methylation is required, but not sufficient for a stable Treg phenotype induction. Therefore, targeted demethylation of the TSDR may be a critical addition to published *in vitro* Treg induction protocols which so far lack FOXP3 stability.

## Introduction

Regulatory T cells (Tregs) harbor immuno-suppressive functions with which they prevent auto-immune reactions and dampen overshooting inflammation. This makes them promising effector cells for antigen-specific adoptive T cell therapies to fight autoimmune diseases, chronic inflammation and complications after organ transplantations, such as graft-versus-host disease and graft rejection reactions (1–4).

Tregs are mainly generated in the thymus (tTregs) as a separate lineage during thymic T cell development (5). They are characterized by the steady-state expression of the IL2 receptor alpha chain (CD25) as well as the lineage transcription factor FOXP3 (6–10). However, conversion of conventional mature T cells to the Treg lineage in the periphery (so-called pTregs) is also possible and has been described for antigens derived from food or commensal microflora (11–13). tTregs and pTregs together form the pool of naturally-occuring Tregs (nTregs) which display a stable immuno-suppressive phenotype with constitutive expression of FOXP3 required for the immuno-suppressive function (14). Stable imprinting of the phenotype has been suggested to be mediated by the Treg-specific de-methylated region (TSDR, also called CNS2) in the FOXP3 gene, an epigenetic switch region which is selectively activated by DNA de-methylation in tTregs and stable pTreg populations and sustains FOXP3 protein expression by epigenetic regulation (15–19). This is in contrast to T cells expressing FOXP3 only transiently, such as recently activated human conventional T cells and *in vitro* TGF-ß-induced Tregs (iTregs) (20, 21). Both of these cell types lack TSDR activation by de-methylation and hence, are prone to lose FOXP3 expression and with it, Treg functionality (16, 17, 19). Their instable phenotype currently excludes iTregs from application in adoptive T cell therapy, although they may harbor selective benefits: they can be generated in large numbers *in vitro* and can be selected for a given antigen-specificity, especially when disease-driving effector/memory populations from patients could be used in an autologous setting. As an additional complication here, TGF-ß-induced iTregs cannot be generated from antigen-experienced memory T cell populations (20, 22, 23).

While the correlation of TSDR de-methylation and stability of FOXP3 expression has been reported by many groups, the selective causal role of DNA methylation on TSDR and its Treg-inducing potential has not been defined yet, despite of its clinical relevance. This was mainly due to technical limitations for the targeted epigenetic editing of switch regions in the endogenous chromatin environment of a living cell. Novel approaches for targeted DNA de-methylation at regulatory elements have recently been developed for such purposes (24–33), however, these approaches await successful implementation in therapeutically relevant primary human T cell subsets. In a study aiming at targeted TSDR de-methylation in primary murine T cells, only small changes in the degree of methylation could be achieved with no observable functional consequences (34).

We present here a powerful “hit-and-run” epigenetic editing approach that induced a complete and lasting DNA de-methylation at the TSDR in primary human T cells. The CRISPR-Cas9-based method allowed us to uncover a causal relationship between TSDR de-methylation and FOXP3 protein expression, with TSDR de-methylation alone being sufficient to induce FOXP3 expression in both naïve and even effector/memory populations. Epigenetic editing of the TSDR induced FOXP3 mRNA in its physiological isoform distribution and protein within its physiological expression limits.

The presented method therefore allows the identification of causal roles of epigenetic states in critical regulatory elements and thorough gain-of-function testing for epigenetically regulated gene products, both of which are of profound scientific interest in cellular and molecular biology.

Published iTreg generation protocols for the application of *in vitro* induced Tregs in therapy are so far hampered by the lack of stability-determining epigenetic modifications of the TSDR. While our study shows that epigenetic editing of the TSDR is possible and may be propagated stably through extensive proliferation, a functional Treg phenotype could not be induced. Thus, a combination of both approaches could represent an important step towards functional and stable Treg products for clinical application. Moreover, epigenetic editing of therapeutic T cell products at other gene loci might offer additional options for optimization with regard to properties such as functional differentiation, stability or longevity which are epigenetically regulated.

## Materials and Methods

### Plasmid Generation

All generated plasmids, their Addgene ID and contained guide sequences are listed in **Supplementary Table 1**. The empty plasmid for targeted DNA de-methylation pSpdCas9-huTET1CD-T2A-mCherry(PX458) (Addgene #129027) and the catalytically inactivated control pSpdCas9-hu**d**TET1CD-T2A-mCherry(PX458) (Addgene #129028) were generated as follows: mCherry was PCR amplified from pICSL50004 (Addgene #50316) using forward 5’GATCgaattcGGCAGGGGAGAGGGCAGAGGAAGTCTGCTAACATGCGGTGACGTCGAGGAGAATCCTGGCCCAGTGAGCAAGGGCGAGGAGGATAAC3’ and reverse 5’ GATCgaattcTTACTTGTACAGCTCGTCCATGCCGCCGGTGGAGTGGCGGCCCTCGGCGCGTTCGTACTGTTCCACGATGGTGTAGTC3’ primers. GFP in pSpCas9(BB)-2A-GFP(PX458) (Addgene # 48138) was excised by EcoRI digestion and mCherry was introduced by ligation. Next, Cas9 was replaced by HA-tag-NLS-dCas9 from pAC91-pmax-dCas9VP64 (Addgene #48223) using AgeI und KflI digestion and religation. Then, the huTET1 catalytic domain and catalytically inactivated dTET1 catalytic domain were PCR amplified from pJFA344C7 (Addgene # 49236) or MLM3747 (Addgene #49965), respectively, using the forward 5’ GATCggccggccAGGGAGGAGGATCCCTGCCCAC 3’ and reverse 5’ GATCggccggccATGACCCAATGGTTA4TAGGGCCCCG 3’ primers and cloned at the C-terminus of dCas9 using FseI digestion and subsequent ligation.

The 20nt guide sequences were first cloned into pSpCas9(BB)-2A-GFP(PX458) (Addgene plasmid # 48138) as described in (35) and subsequently transfered into pSpdCas9-huTET1CD-T2A-mCherry(PX458) (Addgene #129027) or pSpdCas9-hudTET1CD-T2A-mCherry(PX458) (Addgene #129028) using PvuI and XbaI digestion and subsequent ligation.

### Single Guide RNA (sgRNA) Design

Twenty nt guide sequences were designed using Benchling (www.benchling.com) and sequences covering the majority of CpGs in the TSDR with high Off-Target scores (min >50, mostly >70) were chosen.

### Isolation of Human Primary T Cells

Human PBMCs were isolated from peripheral blood from healthy male donors by Ficoll-Paque (GE Healthcare Life Sciences) gradient centrifugation. CD4+ T cells were enriched using CD4 MicroBeads and the Magnetic Activated Cell Sorting (MACS) technology (Miltenyi Biotec). Subsequently conventional naïve T cells (Tnaive, CD3+CD4+CD25neg, CD127+, CD45RA+, CD45ROneg), memory Th1 (CD3+CD4+CD25neg, CD127+, CD45RAneg, CD45RO+, CXCR3+) and regulatory T cells (Treg, CD3+CD4+CD25hi, CD127low) were sorted on a FACSAria II instrument (BD Biosciences). All antibodies used are listed in **Supplementary Table 2**.

### Isolation of Murine Primary T Cells

C57BL/6 mice were bred at the Bundesinstitut fuer Risikobewertung (Berlin, Germany) under SPF conditions and sacrificed at 8–12 weeks of age in agreement with all national and local laws. Pooled erythrocyte-depleted spleen and lymph node cells were stained with anti-mCD25-APC, incubated with anti-APC microbeads (Miltenyi Biotec) and depleted of CD25+ cells using the MACS technology. Next, cells were enriched for CD4+ T cells using CD4 (L3T4) MicroBeads and the MACS technology. All antibodies used are listed in **Supplementary Table 2**.

### Cell Culture

Human Tnaïve and Th1 cells (pooled from 5–6 donors) were cultured in T cell medium [RPMI medium 1640+GlutaMAX (Thermo Fisher) incl. 10% (v/v) FBS (Corning), 100 U/ml penicillin/100 µg/ml streptomycin (Thermo Fisher), 50 µM 2-Mercaptoethanol (Thermo Fisher), 25 mM HEPES-Buffer (Merck), 1 mM Sodium Pyruvate (Merck), 100 µg/ml L-Ascorbic acid (Sigma-Aldrich), 20 ng/ml recombinant human IL-2(rhIL-2, R&D Systems)] and activated for 3 days with plate bound anti-CD3 & anti-CD28 antibodies (**Supplementary Table 2**). Then, cells were harvested, washed with PBS and left untreated or were transfected with the indicated plasmids or MOCK transfected. Cells were cultured 12 h in antibiotic free medium and another 36 h in T cell medium. Then, samples were sorted according to their mCherry expression (mCherry+ “TET1” or mCherry-) and re-cultured until analysis on day 7 post transfection. For long term culture, cells were restimulated on day 9 post transfection and then every 5–7 days with 1 bead per 1 cell using the Treg Expansion Kit (Miltenyi Biotec).

Human Treg (from individual donors) were cultured in Treg medium [TexMACS medium (Miltenyi Biotec) incl. 5% (v/v) human AB-serum (Sigma-Aldrich), 100 U/ml MACS GMP rhIL-2, 100 nM rapamycin (both Miltenyi Biotec) 100 U/ml penicillin/100 µg/ml streptomycin (Thermo Fisher)] and activated with 4 beads per 1 Treg using the Treg Expansion Kit (Miltenyi Biotec).

For clonal expansion culture, mCherry+ “TET1”, mCherry- or MOCK transfected single cells (on day 2 post transfection) were sorted into 96-well round bottom plates containing 50.000 γ-irradiated (3.000 cGy) PBMCs (mix of 3 autologous donors) in Tnaïve sort medium [TexMACS medium (Miltenyi Biotec) incl. 10% (v/v) human AB-serum (Sigma-Aldrich), 100 U/ml penicillin/100 µg/ml streptomycin (Thermo Fisher), 20 µM 2-Mercaptoethanol (Thermo Fisher), 20 ng/ml recombinant human IL-2(rhIL-2, R&D Systems), 1 µg/ml anti-CD28 (Miltenyi Biotec)]. On day 1 post sort, 50.000 Treg Expansion Beads (Miltenyi Biotec) were added to the cells. On day 7, 50.000 γ-irradiated PBMCs (mix of 3 autologous donors) were added. Growing clones were re-plated and expanded in culture medium [TexMACS medium (Miltenyi Biotec) incl. 5% (v/v) human AB-serum (Sigma-Aldrich), 100 U/ml penicillin/100 µg/ml streptomycin (Thermo Fisher), 20 ng/ml recombinant human IL-2 (rhIL-2, R&D Systems)] and reactivated (1 bead per 1 cell Treg Expansion Kit) on day 23–25 post transfection. Cells were harvested between day 23 and 29. Single untreated Treg were sorted and cultured similarly but using Treg sort medium [TexMACS medium (Miltenyi Biotec) incl. 10% (v/v) human AB-serum (Sigma-Aldrich), 100 U/ml penicillin/100 µg/ml streptomycin (Thermo Fisher), 100 U/ml MACS GMP rhIL-2 + 100 nM rapamycin (both Miltenyi Biotec), 20 µM 2-Mercaptoethanol (Thermo Fisher) 1 µg/ml anti-CD28 (Miltenyi Biotec) with addition of 100.000 Treg Expansion beads on day 1 after sort. Growing Treg clones were re-plated and expanded in Treg culture medium.

Murine Treg-depleted CD4+ T cells were cultured in T cell medium (supplemented with 10 ng/ml recombinant mouse IL-2 (rmIL-2,instead of rhIL2) and activated for 2 days with plate-bound anti-mCD3 & anti-mCD28 antibodies (**Supplementary Table 2**). Then cells were harvested, washed with PBS and transfected with the indicated plasmids, MOCK transfected or left untreated. After transfection, cells were activated for another 24 h with plate-bound anti-mCD3 & anti-mCD28 antibodies in antibiotic free medium. After additional 24 h in T cell medium cells were sorted according to their mCherry expression (mCherry+ & mCherry-) and cultured in T cell medium until day 8 post transfection.

Jurkat cells were cultured in Jurkat medium [RPMI medium 1640+GlutaMAX (Thermo Fisher) incl. 10% FBS (Corning), 100 U/ml penicillin/100 ug/ml streptomycin (Thermo Fisher)] and maintained at 0.4*10^6^–1.4*10^6^ cells/ml. Cells were harvested, washed with PBS, and transfected with the indicated plasmids or MOCK transfected. Cells were cultured 12 h in antibiotic free medium, another 36 h in Jurkat medium. Then, cells were sorted according to their mCherry expression (mCherry+ & mCherry-) and taken into culture until analysis.

### Transfection

Cells were transfected in batches of 2*10^6^ cells by electroporation using the Neon Transfection System (Thermo Fisher Scientific). Each batch was resuspended in 100 µl T buffer with the addition of 12 µg single plasmid or equimolar plasmid-mix and subjected to 2 pulses, 1,350 V, 20 ms.

### Suppression Assay

As responder cells, human naïve T cells were sorted from allogenic donors as described above. Responder cells were rested overnight in resting medium (RPMI Medium 1640 + GlutaMAX supplemented with 10% FBS and 100 U/ml penicillin/100 μg/ml streptomycin).

Responder cells were stained with CellTrace Violet Cell Proliferation Kit (Thermo Fisher Scientific) according to manufacturer’s recommendations. Fifty thousand responder cells were mixed with 50,000 suppressor cells (cultured Tregs or mCherry+ “TET1” or mCherry- or MOCK transfected on day 9 post transfection) in a 96-well round bottom plate in resting medium. One hundred thousand Treg Suppression Inspector beads (human, Miltenyi Biotec) were added. As controls, 100,000 responder cells with (“responder only act.”) and without beads (“responder only”) were cultured. On day 5 after seeding (= day 14 after transfection), proliferation of the responder cells was assessed by dilution of the CellTrace dye using flow cytometry (MACSQuant instrument, Miltenyi Biotec).

### Staining and Flow Cytometry

Cells were stained in staining buffer (PBS+0.2 %BSA) in different combinations of antibodies listed in **Supplementary Table 2**.

Zombie Green, Zombie Aqua Fixable Viability Kit (Biolegend) or propidium iodide (Sigma-Aldrich, Schnelldorf, Germany) were used to exclude dead cells.

For CD154 and CD137 expression analysis after short term stimulation, cells were depleted from activating beads and rested for 48 h in resting medium (RPMI Medium 1640 + GlutaMAX, 10% FCS, 100 U/ml penicillin/100, μg/ml streptomycin). Subsequently, cells were activated for 6 h with the Treg Expansion Kit (1 bead per 1 cell) in presence of 1 µg/ml anti-CD40 antibody in resting medium.

For Interferon-γ expression analysis, cells were stimulated with 10 ng/ml phorbol 12-myristate 13-acetate (PMA, Sigma-Aldrich) and 500 ng/ml Ionomycin (Sigma-Aldrich) for 5 h. Ten µg/ml Brefeldin A (Sigma-Aldrich) were added after 30 min stimulation.

For intracellular staining of FOXP3 and interferon-γ (IFN-γ), cells were fixed, permeabilized and stained using the Foxp3/Transcription Factor Staining Buffer Set (eBioscience).

Cells were analyzed on a LSRFortessa instrument (BD Biosciences) and data were analyzed using the FlowJo software (FlowJo, LLC).

### Amplicon TSDR Methylation Analysis

DNA was prepared using the GenElute™ Mammalian Genomic DNA Miniprep Kit (Sigma-Aldrich) according to the manufacturer´s protocol. Up to 200 ng genomic DNA or all DNA obtained from pellets containing min. 400 cells was bisulfite-converted using OPTI-Bisulfite (36) or EZ-DNA methylation Gold kit (Zymo, Irvine, USA). Subsequently PCRs were performed (human: 2-5 µl of bisulfite-treated DNA, 1xBD buffer (Solis Biodyne, Tartu, Estonia), 0.25 mM of each dNTP, 0.25 mM MglCl_2_, 2.5U HotFirePol (Solis Biodyne) - murine: 10 µl bisulfite-treated DNA, 80 mM Tris-HCL, 20 mM (NH_4_)_2_SO_4_, 0.2% Tween-20, 2.5 mM MgCl_2_, 0.2 mM of each dNTP, 2.5U HotFirePol) using 0.5 pmol of primers (human_F:5’TTGTTTGGGGGTAGAGGATTTAG-3’, human_R:5’CCTAATATTATACTATTTAAAAACCCC-3’) (murine: 5 pmol of each primer murine_F: 5´-GGGTTTTTTTGGTATTTAAGAAAGAT-3´, murine_R: 5´-AAATCTACATCTAAACCCTATTATCACA-3) with Illumina compatible universal adaptor sequences attached at the 5´-end. PCRs were performed in a thermocycler starting with 15 min 95°C followed by 45 cycles 95°C 1 min, 56°C 2 min (murine 52°C), 72°C 1 min and a 10 min final extension at 72°C. Amplicons were purified with MagSi-NGS Prep Plus beads (Steinbrenner, Wiesenbach, Germany), diluted, pooled and sequenced on the MiSeq platform (v3 chemistry: 2x300 bp paired-end, Illumina, San Diego, USA) following the manufacturer’s instructions aiming at 10,000 reads per amplicon. Reads were aligned and evaluated using the semi-automated tool BiQ-Analyzer HT (37). Read pattern maps were generated in R using in-house scripts.

### Infinium Methylation EPIC Arrays

Genome wide methylation profiles were generated on the EPIC infinium arrays (Illumina, San Diego, USA) as described previously (38). The EPIC array interrogates 865,859 methylation sites covering 95% of CpG islands, 98% of RefSeq genes together with high coverage of FANTOM5 enhancers and ENCODE open chromatin and transcription factor binding sites.

Samples were bisulfite converted using the EZ-DNA methylation Gold Kit (Zymo) and hybridized to the arrays following the manufacturer’s instructions. Arrays were scanned on the HiScan platform (Illumina) and raw data was processed in R with the RnBeads library package [v2.0, (39)] using “Dasen” normalization (40) and greedycut filter (0.05 threshold). Then for each CpG site a beta-value was calculated representing the fraction of methylated cytosines at that particular site (0 = unmethylated, 1 = fully methylated). Beta values were exported from RnBeads for downstream analysis which was performed in R using in-house scripts involving the following library packages: ggplot2 (41), ggfortify (42), reshape2 (43), GenomicRanges (44).

### RNA Sequencing

Total RNA was isolated from snap frozen cell pellets with TRI reagent and the direct-ZOL RNA Miniprep kit (both Zymo) with on-column DNAseI treatment according to the manufacturer’s instruction. Eluted RNA was quantified on a Nanodrop 2000 (ThermoFisher Scientific; Waltham, USA) and integrity was checked on a Bioanalyzer (Agilent; Santa Clara, USA). Samples with a RIN value >9 were used for mRNA library preparation. One hundred ng total RNA were denaturated together with 0.5 µl 20 mM dNTPs and 0.5 µl 20 µM oligo dT primer (5’-AAGCAGTGGTATCAACGCAGAGTACT[30x]VN-3’) by heat for 3 min at 72°C and immediately put on ice, then reversely transcribed in a 10 µl reaction with 0.5 µl SuperScript II reverse transcriptase (ThermoFisher Scientific), 0.4 µl RNAsin (Promega; Madison, USA), 2 µl 5x Superscript II first strand buffer, 0.5 µl 100 mM DTT, 2 µl 5 M Betaine, 0.6 µl 0.1 M MgCl_2_ and 0.5 µl template switch oligo (5’-AAGCAGTGGTATCAACGCAGAGTACATrGrG+G-3’) for 90 min at 42°C followed by 10 cycles (2 min at 50°C, 2 min at 42°C) and a final inactivation step (15 in at 72°C). The cDNA was then pre-amplified with 12.5 µl of 2x KAPA HiFi HotStart Ready Mix (Roche, Basel, Switzerland), 0.25 µl 10 µM IS Primer (5’- AAGCAGTGGTATCAACGCAGAGT-3’), and 2.5 µl nuclease-free water in a thermos cycles with the following program: 98°C for 3 min, 8 cycles of 98°C for 20 s, 67°C for 15 s, 72°C for 6 min, and a final extension step (72°C for 5 min). Next, cDNA was purified with Agencourt AMPure XP beads (Beckman Coulter, Brie, USA) and checked on the bioanalyzer and then tagmented with DNA tagmenting enzyme 1 from the Illumina Nextera library preparation kit (1 µl of enzyme with 8 ng of cDNA) for 10 min at 55°C. Tagmented cDNA was purified with MinElute kit (Qiagen, Hilden, Germany) and amplified in a thermocycler with 2x NEBNext high-fidelity mastermix using the following program: 72°C 5 min, 98°C 30 s, then 11 cycles of 98°C 10 s, 63°C 30 s, 72°C 1 min and a final extension step (72°C 5min). The obtained library was purified with AMPure beads, quantified on a Qubit system (ThermoFisher Scientific) and fragment distribution was checked on a bioanalyzer chip.

All libraries were quantified with the NEBNext Library Quant Kit for Illumina (New England Biolabs), hybridized to a V3 single read flow cell (Illumina) and sequenced for 1x 100 bp reads on a HiSeq2500 machine (Illumina).

Reads were trimmed for adapter contamination and low-quality ends (Q < 20) with the cutadapt-wrapper TrimGalore! [https://github.com/FelixKrueger/TrimGalore] after which they were mapped with the IHEC reference pipeline grape-nf (https://github.com/guigolab/grape-nf). For this we used the hs38 reference genome and gene models from Gencode (v22).

### Computational Analysis

RNA expression values were imported for analysis with DESeq2 (45) with tximport (https://f1000research.com/articles/4-1521/v1). Variance stabilized values were generated with the *vst* function and used for PCA and heatmap figures.

To check for enrichment/depletion of FOXP3 binding sites (bs) in DNA methylation data, the annotation of FOXP3bs in the human genome was taken from (46) and the coordinates were converted from hg18 to hg19 assembly with the UCSC lift-over tool (https://genome.ucsc.edu/cgi-bin/hgLiftOver). The obtained regions were extended by 1 kb up- and downstream and all CpGs featured on the EPIC array within these genomic windows were considered.

To check for changes in DNA methylation at potential sgRNA off-target sites the top 50 in silico predicted of target regions for each individual sgRNA were used (sgRNA design see above). CpGs present on the EPIC array located up to 1 kb up- or downstream from these regions were considered.

Enrichment/depletion of CpGs subsets (FOXP3bs, sgRNA off-targets) in the DNA methylation data was assessed with fisher exact tests.

PCAs were calculated in R using the prcomp function. The top 5,000 CpGs associated with PC1 or PC2 were extracted and annotated to genes. Hits per individual gene were counted and a fisher exact test was run to correct for the total number of EPIC probes for each gene. Obtained p-values were corrected for multiple testing (FDR).

## Results

### Induction of TSDR De-Methylation Is Sufficient to Induce FOXP3 Protein Expression

**Figure 1:**
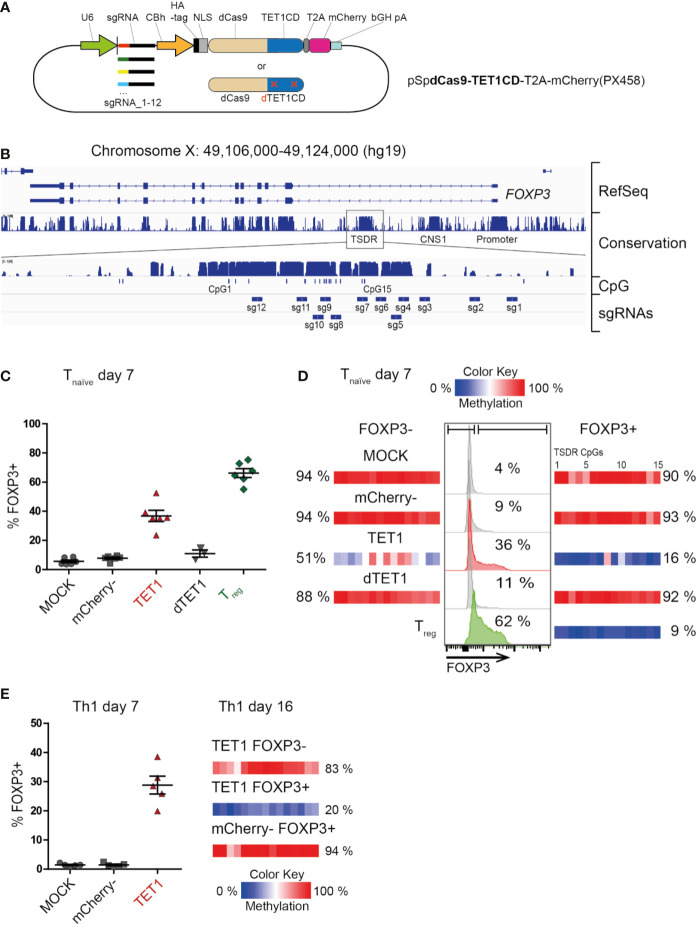
Successful dCas9-TET1CD-mediated targeted de-methylation of the TSDR leads to FOXP3 expression. Ex vivo isolated human naïve CD4+ T cells (T_naive_) or Th1-memory T cells were transfected by electroporation with (or without “MOCK”) a mixture of 12 plasmids **(A)**, each carrying the dCas9-TET1CD fusion protein “TET1” (or the enzymatically dead dCas9-dTET1CD control), one out of 12 single-guide RNA (sgRNA1-12) and a mCherry reporter gene. On day 2 post transfection, cells of the dCas9-TET1CD- transfected sample were sorted by FACS according to mCherry expression (mCherry+ = “TET1” and “mCherry-”). mCherry+ cells of the dCas9-dTET1CD transfected sample were also sorted and used as controls. All samples were re-cultured and analyzed for TSDR methylation and FOXP3 expression at the indicated time-points. Ex vivo sorted CD4+ CD25hi CD127- Treg were cultured for the same time period, left untransfected, and used as positive controls. **(B)** The highly conserved TSDR in the human FOXP3 locus is shown and the positions of the CpGs as well as the targeted regions for each sgRNA are indicated. **(C)** Fraction of FOXP3+ cells in the indicated sample groups as assessed by intracellular FACS staining on day 7 post transfection of CD4+ naïve T cells, n = 6, for dTET1 n = 3, the mean and SEM are indicated. **(D)** Representative histograms of FOXP3 expression in naïve T cell-derived experimental groups (MOCK, mCherry-, TET1 and dTET1) on day 7 post transfection (middle, the fraction of FOXP3+ cells is given in %). Untransfected ex vivo-sorted Treg function as a positive control. On the same day, all populations were sorted into FOXP3- and FOXP3+ fractions. The degree of TSDR methylation after re-sorting (left: FOXP3-, right: FOXP3+) is shown (color scale) for each of the 15 TSDR-CpGs (small boxes). The mean methylation degree of the entire TSDR is given in %. **(E)** left: Expression of FOXP3 in Th1 memory T cells on day 7 post transfection (the mean and SEM are indicated). right: The degree of TSDR methylation of FOXP3+/- resorted populations and control cells on day 16 is indicated, for each TSDR CpG individually (small boxes with color scale) and as the mean of all (in %).

To establish targeted de-methylation of epigenetic control elements in primary human T cells, we generated a plasmid coding for the enzymatically-dead Cas9 protein (dCas9) coupled to the catalytic domain of the human TET1 enzyme (dCas9-TET1CD, **Figure 1A**). This editing complex still harbors the target binding capacity of the CRISPR-Cas9-system, but does not introduce double-strand breaks into the DNA, since the catalytic domain of the Cas9 enzyme has been mutated. Instead, the coupled TET1CD will oxidize methylated CpG-motifs in the vicinity of the targeted binding site and with this, start the DNA de-methylation cascade in a locus specific manner. The plasmid also carried a mCherry reporter and a single-guide RNA (sgRNA) targeting the human TSDR (**Figure 1A**). We generated 12 plasmid variants each carrying 1 of 12 unique sgRNAs which, together, would cover the majority of the TSDR (**Figure 1B**). A similar set of plasmids, but coding for an enzymatically-dead TET1 protein (dCas9-dTET1CD), was generated as a control (**Figure 1A**).

Pools of all 12 dCas9-TET1CD or control plasmids were transiently transfected into ex vivo isolated human naïve Treg-depleted CD4+ T cells (Tnaive, CD3+CD4+CD25-CD127+CD45RA+CD45RO-) which were isolated by FACS from peripheral blood of healthy male donors and activated via CD3/CD28 for three days. Successfully transfected mCherry+ T cells (“TET1”) and mCherry- controls were re-sorted two days later and re-cultured. On day 7 post transfection, TET1 samples displayed a markedly increased expression of FOXP3 protein (**Figures 1C, D**) compared to mCherry-, successfully transfected dTET1 and mock-transfected control (MOCK) samples. This correlated to a strongly reduced TSDR methylation level in the TET1, but not in the control samples (**Supplementary Figure 1**). Sorting of the samples according to FOXP3 protein expression confirmed that the FOXP3+ TET1 cells displayed complete TSDR de-methylation in contrast to the MOCK, mCherry- and dTET1 controls (**Figure 1D** and **Supplementary Figure 1**). TET1 cells not expressing FOXP3 (FOXP3- TET1) also displayed a reduced mean TSDR methylation degree (51%). However, their DNA methylation pattern (**Supplementary Figure 1**) revealed that the vast majority of cells (captured in individual reads in **Supplementary Figure 1**) displayed heterogeneous methylation patterns of CpGs within the TSDR, while in FOXP3+ TET1 cells, most of the cells displayed de-methylation of most or all CpGs within the TSDR, indicating that extensive de-methylation is required to facilitate TSDR-mediated FOXP3 expression. This goes in line with results from a murine study, were induction of heterogeneous demethylation patterns at the TSDR did not result in functional activation of the TSDR (34).

These results demonstrate that successful targeted DNA de-methylation of the TSDR can be achieved and is sufficient to induce FOXP3 expression. To our knowledge, this is the first functional proof of a causal relationship between TSDR de-methylation and FOXP3 expression in living human T cells. Importantly, targeted TSDR de-methylation could also be achieved in fully differentiated, ex vivo isolated pro-inflammatory memory T cells (Th1-type: CD3+CD4+CD25-CD127+CD45RA-CD45RO+CXCR3+; **Figure 1E**). This demonstrates that epigenetic editing has the power to overcome preformed lineage specification (here: Th1), as expression of master transcription factors of opposing functional lineages (here: Treg) have been reported to be epigenetically silenced (47). Also, it enabled FOXP3 induction in (non-Treg) memory T cells, which was not possible using TGF-ß treatment (20, 22, 23). We also tested TSDR epigenetic editing in murine Tnaive cells as Foxp3 expression in mice has been reported to be more rigidly controlled compared to human T cells (48), since human but not murine conventional T cells transiently upregulate FOXP3 as part of the normal activation process in the absence of TSDR de-methylation (7, 49, 50). Indeed, induced complete TSDR de-methylation in murine cells resulted in Foxp3 protein expression in Tnaive cells (**Supplementary Figure 2**), further supporting the causality between TSDR de-methylation and FOXP3 protein expression and demonstrating the powerful modifying capacity of the dCas9-TET1CD complex.

### Targeted TSDR De-Methylation Is Locus Specific and Results in a Partial Remodeling of the DNA Methylome

**Figure 2:**
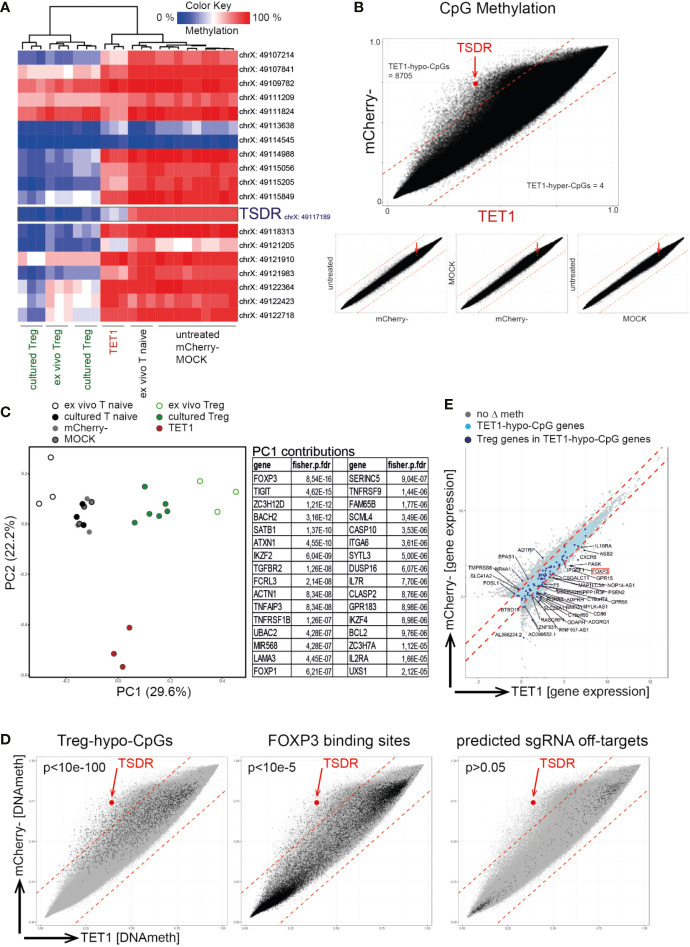
Targeted TSDR de-methylation results in a partial remodeling of the DNA methylome towards a Treg signature. Successfully transfected mCherry+ naïve T cells (“TET1”) on day 7 post transfection were analyzed for their genome-wide DNA methylation profile using the 850K Illumina EPIC bead-chip Array and compared to ex vivo isolated and cultured Treg (positive controls) as well as to several negative controls [ex vivo naïve T cells (“Tnaive”), untreated cultured Tnaive, MOCK treated and mCherry- cells]. **(A)** Hierarchical clustering of analyzed samples (n = 3) based on the methylation degree of all CpGs within the FOXP3 locus (genomic locations indicated). The TSDR is highlighted. **(B)** Correlation plots of all EPIC Array CpGs for the indicated samples (mean of n = 3). The TSDR is highlighted (red dot and arrow), the numbers of TET1-hypo- and TET1-hyper-methylated CpGs are indicated. **(C)** left: PCA of EPIC Array DNA methylation data. right: The top 32 genes enriched with differentially methylated CpGs contributing to PC1 are listed (based on adjusted fdr values). **(D)** Correlation plot of TET1 vs. mCherry- samples (as in B) with highlighted selections of CpGs. left: Treg-hypo-CpGs (cultured Treg vs. cultured untreated naïve T cells, Δ ≥ 0.2), middle: FOXP3 binding sites (46), right: predicted off-targets of the 12 sgRNAs. p-values for enrichments within TET1-hypo-CpGs are indicated (fisher exact test). **(E)** Correlation plot of gene expression values (from RNAseq) in TET1 vs mCherry- samples. Genes enriched with TET1-hypo-CpGs are indicated in light blue or in dark blue in case they are also differentially expressed in Treg vs. Tnaive (“Treg genes”). The names of the Treg genes which are enriched with TET1-hypo-CpGs and displaying differential expression in TET1 samples are given.

We next assessed the specificity of the epigenetic editing process using genome-wide DNA methylation screening (Infinium Methylation EPIC 850K Array). Within the FOXP3 locus, only the TSDR-CpG displayed selective de-methylation in TET1 cells while all other tested FOXP3 CpGs behaved comparable to the negative controls (**Figure 2A**). Global comparison of the methylome displayed high concordance between the biological replicates (**Supplementary Figure 3**), and revealed a selective de-methylation (Δ ≥ 0.2) of approx. 1% of all CpGs (8,705 CpGs, “TET1-hypo-CpGs”) in the TET1 samples compared to mCherry- (**Figure 2B**). Principle component analyses (PCA) of the genome-wide methylomes displayed a shift of the TET1 sample towards the Treg controls on the most important principle component (PC) 1 (**Figure 2C**). The list of genes found to be enriched with differentially methylated CpGs contributing to PC1 confirmed that PC1 is representative of Tnaive-to-Treg development as it contained many known Treg signature genes (e.g. FOXP3, TIGIT, SATB1, IKZF2, TNFRSF9, IKZF4, IL2Ra; **Figure 2C**). This indicated that the edited TET1 cells have started to remodel their epigenome towards a Treg-like signature. Furthermore, TET1-hypo-CpGs showed a significant enrichment for Treg-specific hypomethylated CpGs (“Treg-hypo-CpGs”; cultured Treg vs. Tnaive, Δ ≥ 0.2) as well as for CpGs located at reported FOXP3 binding sites (46) (**Figure 2D**, left and middle) further supporting the interpretation of a partially-induced Treg epigenome. In contrast, no enrichment for predicted sgRNA off-targets could be detected (**Figure 2D**, right). Finally, a fraction of TET1-hypo-CpG-associated genes displayed differential expression in TET1 vs. mCherry- cells (**Figure 2E**, light blue dots) of which 37 genes were also differentially expressed in Tregs (“Treg genes”; **Figure 2E**, dark blue dots), indicating that the induced epigenetic switch leads to the induction of selected Treg signature genes.

### TSDR-Induced FOXP3 Expression Does Not Induce a Functional Treg Phenotype

**Figure 3:**
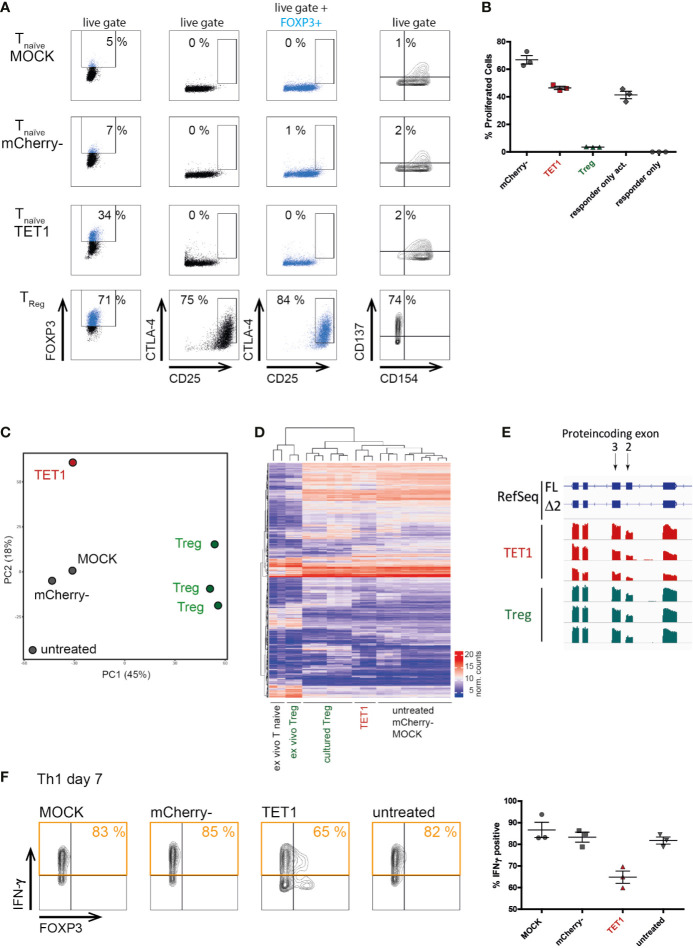
Physiological FOXP3 expression induced by targeted TSDR de-methylation does not convert conventional T cells into functional Tregs. Successfully transfected mCherry+ naïve T cells (“TET1”) on day 7 post transfection were analyzed for their surface protein expression, transcriptome and for their Treg function and compared to positive [ex vivo Treg and cultured Treg] as well as to negative controls (mCherry-, MOCK, untreated). **(A)** Expression of the known Treg markers FOXP3, CD25 and CTLA-4 in the indicated samples (black) or pre-gated on FOXP3+ cells (blue). Expression of CD137 and CD154 were stained after additional 2 days of rest and subsequent short-term TCR re-stimulation. One representative experiment of 3 is shown for each staining. **(B)** In vitro suppression assay to assess the suppressive capacity of TET1 and control cells on day 9 post transfection. The fraction of proliferating CD4+ responder cells in a co-culture system using the indicated populations (X-axis) as suppressor cells is given. The mean and SEM are indicated. One representative experiment of 2 is shown. **(C)** PCA of genome-wide transcriptomes (RNAseq). Shown is one representative experiment of n = 3. **(D)** Hierarchical clustering of samples from all three experiments based on the top 1000 differentially expressed genes. **(E)** Screenshot of a genome-browser view displaying FOXP3 expression in TET1 and Treg samples (n = 3 each). The coding exons 1–5 are shown to compare the relative fractions of the two annotated FOXP3 isoforms, full length (FL) and Δ exon 2 (Δ 2) in both cellular subtypes. **(F)** Interferon γ expression after short-term re-stimulation with PMA/ionomycin in transfected TET1 Th1 memory cells and controls. One representative experiment is shown in FACS plots, results of n = 3 are shown in the graph on the right, the mean and SEM are indicated.

Next, we assessed whether epigenetic remodeling resulted in a full functional switch towards a Treg-like phenotype. We assessed the expression of classical Treg markers (CD25, CTLA4) and the activation-induced CD137/CD154 Treg-phenotype (51, 52). Surprisingly, TET1 cells resembled the negative controls in these assays and did not upregulate Treg markers (**Figure 3A**). Similarly, in *in vitro* suppression assays, TET1 cells did not display suppressive capacity compared to the activated responder-only controls (**Figure 3B**), indicating that a characteristic suppressive Treg function was not induced by epigenetic editing of the TSDR. However, TET1 cells did not support enhanced responder proliferation, as the mCherry- controls did, indicating that they still displayed functional alterations as a response to the editing treatment. Additionally, transcriptomic profiling (RNAseq) placed the TET1 cells in proximity to the negative controls in both, PCA (**Figure 3C**) and in hierarchical clustering (**Figure 3D**), indicating that the RNA expression profile in general has not been switched to a Treg-like signature. In contrast to classical gain-of-function-experiments using ectopic overexpression, our system of epigenetic editing mediates expression from the endogenous locus and hence, is subject to physiological regulation. This is visible in the resulting physiological splice-isoform distribution, which in edited TET1 cells was comparable to the Treg controls (**Figure 1D** and **Figure 3E**, respectively). These results indicate that induction of physiological FOXP3 expression in naïve CD4+ T cells is not sufficient to induce a Treg-like phenotype, in contrast to previous reports using ectopic overexpression systems (8, 9, 53). However, epigenetically edited Th1-Tmem cells displayed a reduced expression of the pro-inflammatory signature cytokine IFN-γ (**Figure 3F**).

While TSDR de-methylation alone might not be sufficient to induce functional Tregs, it is likely that simultaneous editing of several known epigenetic Treg regulators (54) could be successful. To assess the technical feasibility of such an approach, we investigated whether targeting of the TSDR using several sgRNAs covering the entire region was required or whether individual sgRNAs would suffice for productive de-methylation of the complete TSDR enhancer. For this, each plasmid coding for dCas9-TET1CD and one sgRNA was transfected into Jurkat cells individually (or as a pool as a reference), to screen for their editing potential. The 12 sgRNAs induced very different de-methylation patterns ranging from weak effects on all CpGs, to rather selective effects on individual CpGs, to strong effects throughout the TSDR (**Supplementary Figure 4**). No obvious correlation between the target-site location of the sgRNA and the affected CpGs within the TSDR could be observed. SgRNA8 and sgRNA7 displayed the greatest potential, which was almost comparable to the sgRNA pool, indicating that even individual sgRNAs can be sufficient to modify entire regulatory elements in epigenetic editing approaches. This paves the way for a potential simultaneous editing of several target regions for Treg induction.

### Induced TSDR De-Methylation Can Be Stably Maintained

**Figure 4:**
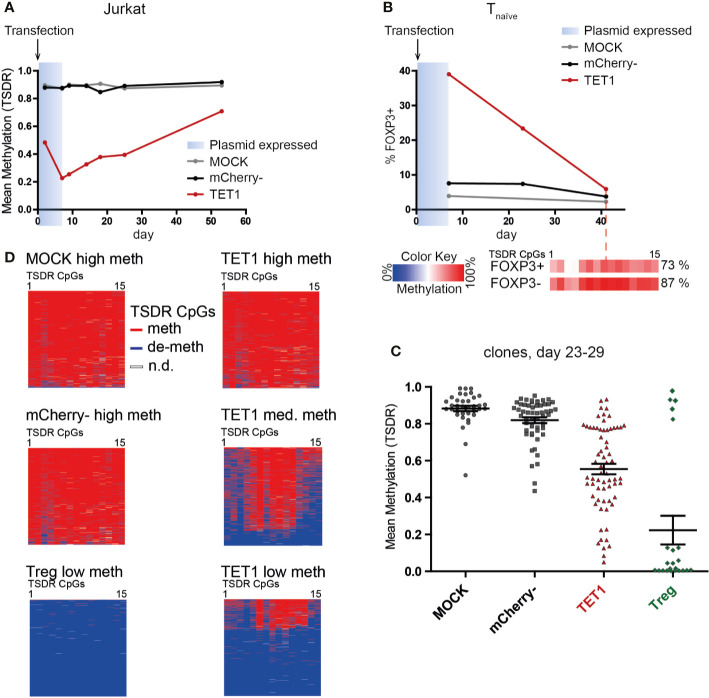
Efficient TSDR de-methylation can be stably maintained during proliferation even after expression of dCas9-TET1CD has ceased. **(A)** Kinetics of TSDR methylation in TET1, mCherry-, and MOCK Jurkat cells. Jurkat cells were analyzed for TSDR methylation at the indicated time-points post transfection (shown is one representative experiment of two). The estimated time-frame of dCas9-TET1CD plasmid expression is indicated in blue. **(B)** Samples of TET1, mCherry-, and MOCK naïve T cells were analyzed for FOXP3 expression at several time-points post transfection (shown is one representative experiment of two). The estimated time-frame of dCas9-TET1CD plasmid expression is indicated in blue. On day 42, the TSDR methylation level in the remaining FOXP3+ and FOXP3- fraction was analyzed (methylation levels for each CpG are indicated in boxes according to the color scale). The mean TSDR methylation level is indicated in percent. **(C)** On day 2 post transfection, single cells of TET1, mCherry-, and MOCK naïve T cells and untreated Tregs were resorted from each group as founder cells for clonal cultures. TSDR methylation levels of the grown clonal cultures 23–29 days post transfection were analyzed. Each dot represents a clonal culture, the mean and SEM are indicated. Pooled results from 2 independent experiments are shown. **(D)** The TSDR methylation patterns obtained from Amplicon-seq for selected examples of the generated clones in C are shown. The results are shown for each analyzed read (lines) and each of the 15 TSDR CpG within the Amplicon (columns).

After successful epigenetic editing, we wanted to assess the stability of the induced TSDR de-methylation, as a reversion of the induced state induced by the surrounding original chromatin environment might occur. In addition, long-term stability of the induced state is an important prerequisite for a potential future application of epigenetic editing in adoptive cellular therapy. Therefore, we tested the stability of the induced TSDR de-methylation after expression of the transiently transfected dCas9-TET1CD has ceased (approx. day 7). TET1 transfected Jurkat cells displayed pronounced TSDR de-methylation as early as day 2 post transfection with a peak at day 7 (**Figure 4A**). Although the induced de-methylated state was clearly maintained beyond day 7, the TET1 Jurkat population displayed an increasing TSDR methylation level with long-term culture (**Figure 4A**). Similarly, TET1 transfected Tnaive cells progressively lost FOXP3 expression during expansion culture and presented a highly methylated TSDR on day 42 with background FOXP3 expression levels (**Figure 4B**). Stability assessment in heterogeneous bulk cultures, however, is confounded by the possibility of out-growth of small (unmodified) populations. This is of particular importance here, as FOXP3 expression has been described to reduce the proliferation capacity of T cells (55). Therefore, we sorted single cells shortly after transfection and generated clonal cultures to prevent selective survival and proliferation competition. Indeed, by this approach we observed some fully de-methylated TET1 transfected clones after 3–4 weeks of expansion culture (**Figure 4C**). This result indicated that induced TSDR de-methylation can be stably maintained by the physiological epigenetic maintenance machinery even during strong proliferation *in vitro*. We assume that in this case, the original founder cell was already strongly or even completely de-methylated at the time-point of the single-cell sort (day 2 post transfection). However, most founder cells at the day of sorting probably have achieved only a partial de-methylation and hence, continued to de-methylate the TSDR erratically in their progeny as long as the editor complex was still expressed. This resulted in heterogeneous methylation patterns in the clonal cultures on the day of analysis (day 23–29, **Figure 4D**, “TET1 med meth” and “TET1 low meth”), accumulating to a mean intermediate TSDR methylation level (30–80%, **Figure 4C**). These results indicate that induced TSDR de-methylation can be stably maintained, but the timing and experimental conditions for this imprinting still have to be determined.

## Discussion

Our results demonstrate that our transient-transfection approach of CRISPR-dCas9-TET1-mediated targeted DNA de-methylation is a powerful tool to selectively change the epigenetic code in a target region of interest in fully differentiated living primary human T cells. With this, the determination of causal relationships between regulatory elements and expression control of the regulated gene becomes feasible, as we have shown here for the well-known Treg enhancer TSDR and its associated gene FOXP3. Activation of the TSDR by induced demethylation was sufficient to induce FOXP3 protein expression in primary human T cells, providing a so-far missing causal link between TSDR activity and FOXP3 induction. Importantly, these kind of functional studies assess the regulatory element directly in its endogenous chromatin context, which takes long range interactions, the physiological transcription factor network, counter-regulating features and downstream remodeling into account and at the same time allows expression control of the regulated gene only within its physiological limits. This ensures adequate gain-of-function testing in contrast to ectopic overexpression approaches, which is an important step not only for basic research but also for the identification of promising target regions for the optimization of therapeutic T cell products for adoptive T cell therapy.

For FOXP3 we here show that TSDR-mediated FOXP3 induction is not sufficient to convert a conventional naive CD4+ T cell into a fully functional Treg. This is surprising as retroviral overexpression of FOXP3 has been reported to confer suppressive function to conventional T cells (9, 53). However, in this scenario, a forced ectopic overexpression of FOXP3 occurs, in contrast to physiological FOXP3 induction in our epigenetic editing approach. On the other hand, our results are in line with the notion that the FOXP3 protein requires a preformed Treg-specific epigenetic landscape to act on and fulfill its function as the Treg lineage transcription factor (13, 56, 57). Such an epigenetic profile is imprinted during thymic development in tTregs and its formation precedes expression of FOXP3 protein (56, 58). The lack of a Treg epigenome is also the reason why transient activation-induced FOXP3 expression in conventional T cells and TGF-ß-mediated FOXP3 expression in iTregs does not result in the formation of an nTreg transcriptome (57).

While TSDR-mediated FOXP3 expression has proven to be insufficient on its own to induce Treg function, the targeted epigenetic editing of TSDR might be combined with known iTreg-induction protocols (20, 21), which so far lack epigenetic imprinting (15–17). This would be a significant improvement to the reported approaches, such as *in vitro* TGF-ß treatment together with vitamin-C, which was shown to induce TSDR de-methylation in mice due to enhanced TET activity, but only induced little de-methylation in primary human naive T cells (59). In vitro Foxp3 induction in memory T cells using a small molecule inhibitor for CDK8/19 has recently been reported, however, without induction of TSDR de-methylation (60). Such converted memory T cells could be a promising therapeutic in diseased situations, where pro-inflammatory T cells are clonally expanded but antigen specific Tregs are lacking, as found in allergy to aero-antigens (61). Furthermore, antigen-specific Tregs showed an increased therapeutic potential compared to polyclonal Tregs, e.g in animal models for type 1 diabetes (T1D) (62, 63) or organ transplantation (64, 65). Antigen-specific effector/memory T cells can be isolated from the patient for possible conversion into stable, antigen-specific regulatory T cells by targeted TSDR demethylation. Additionally, possible remaining expression of lineage transcription factors or homing receptors from the original pro-inflammatory cell (e.g. T-bet and CXCR3 for Th1 cells) might further tailor the generated Tregs to superior suppression of the original pro-inflammatory T cells as T-bet+ and CXCR3+ regulatory T cells were shown to be able to migrate to the site of Th1 inflammation enabling local suppression (66–68).

Epigenetic editing of various regulator regions might be used to further optimize a generated Treg population for better homing, survival, suppressive function or stability. We show that this is feasible using our transient “hit-and-run” dCas9-TET1CD approach as an entire enhancer was successfully modified using a single sgRNA and simultaneous transfection of multiple sgRNAs was possible. In addition, the induced epigenetic switch was stably maintained even during strong expansion and after expression of the epigenetic editor has ceased, making off-target-prone continuous overexpression obsolete. We expect that this and similar systems of targeted epigenetic editing may be utilized in the future for adoptive T cell therapy approaches, during which T cells are expanded *in vitro*, creating a window of opportunity for targeted epigenetic improvements to the T cell product without the burden of implementing an *in vivo*-delivery system.

## Data Availability Statement

RNA-Sequencing data are deposited with the European Genome-phenome Archive (EGA) under accession number EGAS00001004867, considering the data protection rights of the human donors. Generated plasmids (Addgene IDs: 129027-129064) are deposited with Addgene and verified sequeces can be found at www.addgene.org. Other original data presented in this study can be obtained upon request to the corresponding author.

## Ethics Statement

The studies involving human participants were reviewed and approved by Ethikkomission der Charité, votes EA1_221_18, EA1/116/13 and EA1/095/13. The patients/participants provided their written informed consent to participate in this study. 

## Author Contributions

CK, DH, and CD performed functional T cell analyses. GG and ST performed bisulfite-sequencing and RNA-sequencing. GG, KN, AS, and ST performed computational analyses. CK, PR, H-DV, JW, AH, and JP designed and coordinated the study. CK, AH, and JP wrote the manuscript with contributions from PR, H-DV, JW, and other authors. All authors contributed to the article and approved the submitted version.

## Funding

This study was funded by the Deutsche Forschungsgemeinschaft (PO2058/1-1), the Leibniz Gemeinschaft (K59/2017), the European Research Council (EpiTune, ERC Starting grant 2018) and the ReShape Horizon 2020 program (no. 825332), all to JP. KN and AS were supported by the German Federal Ministry of Research and Education grant for de.NBI (031L0101D). KN is now employed by AstraZeneca.

## Conflict of Interest

The authors declare that the research was conducted in the absence of any commercial or financial relationships that could be construed as a potential conflict of interest.
