# Supplementary material for: Targeted De-Methylation of the FOXP3-TSDR Is Sufficient to Induce Physiological FOXP3 Expression but Not a Functional Treg Phenotype
Source: Front Immunol. 2021 Jan 7;11:609891. doi: 10.3389/fimmu.2020.609891 (PMC7817622; doi:10.3389/fimmu.2020.609891)
Supplement: Supplementary file 1 [file DataSheet_1.pdf]

Supplementary Materials to

Targeted demethylation of the FOXP3-TSDR is sufficient to induce physiological FOXP3 expression but not a functional Treg phenotype.

Christopher Kressler[1,2], Gilles Gasparoni[3], Karl Nordström[3], Dania Hamo[1,2], Abdulrahman Salhab[3], Christoforos Dimitropoulos[2], Sascha Tierling[3], Petra Reinke[1,4], Hans-Dieter Volk[1,4], Jörn Walter[3], Alf Hamann[2] and Julia K. Polansky[1,2,*]

Supplementary Figures and Tables:

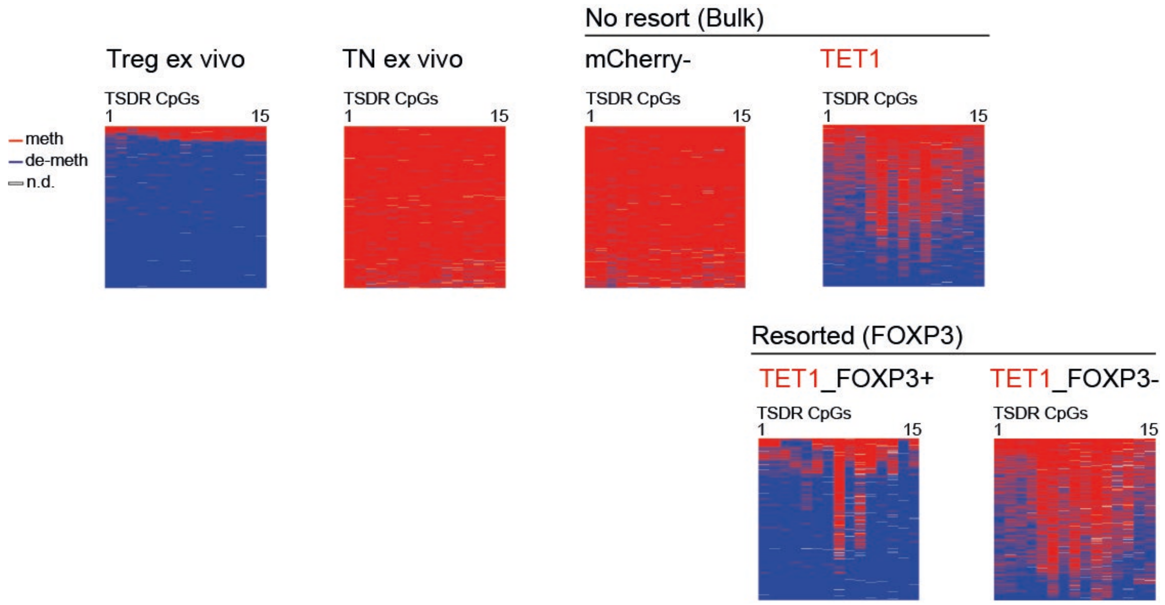

Supplementary Figure 1: Representative TSDR methylation patterns of transfected (FOXP3+ and FOXP3-) TET1 naïve T cells on day 7 post transfection and ex vivo Treg and T naïve control cells. The results of Amplicon-based bisulfite-sequencing are shown for each analyzed read (lines) and each of the 15 TSDR CpG within the Amplicon (columns).

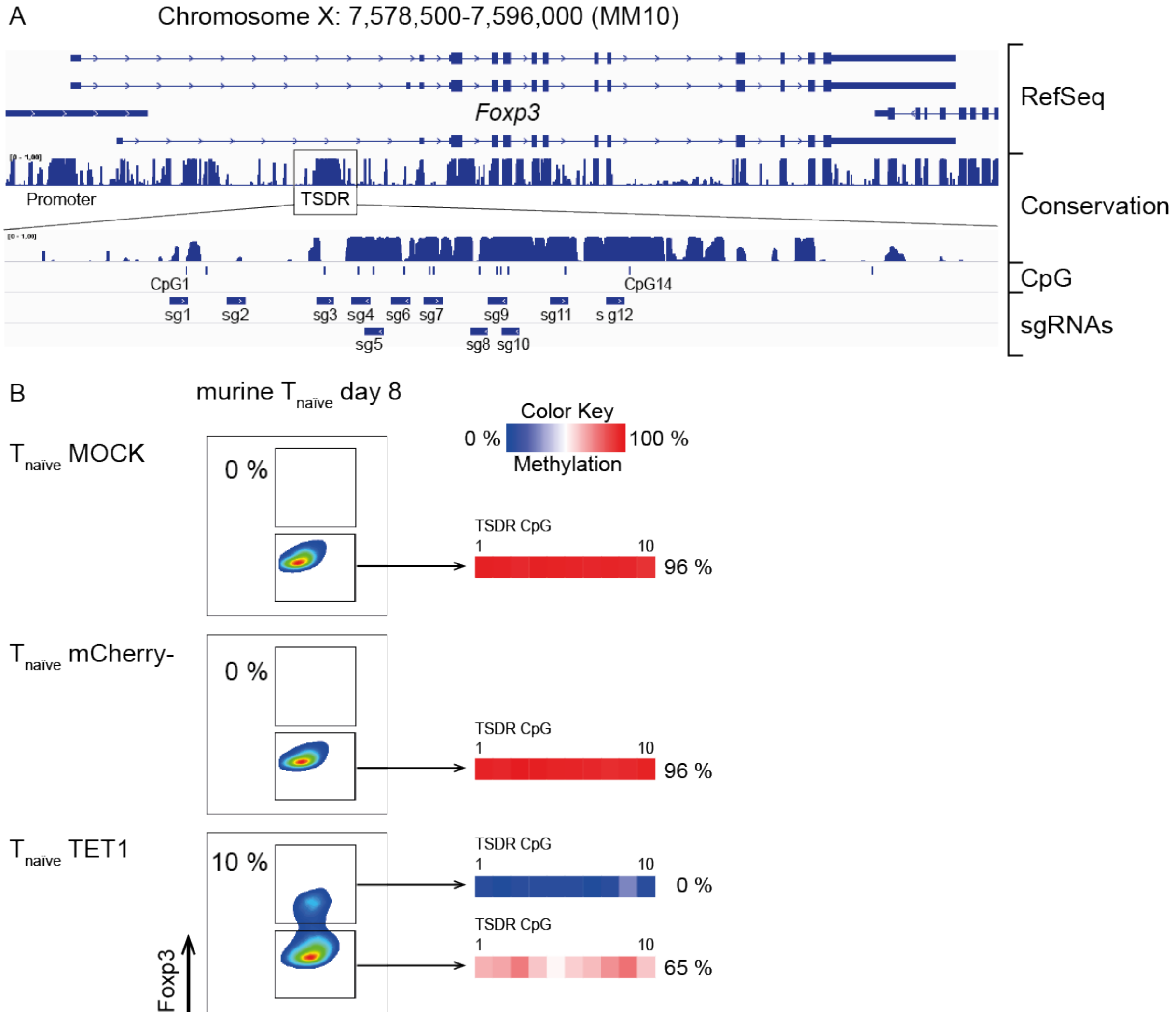

Supplementary Figure 2: Successful dCas9-TET1CD-mediated targeted TSDR demethylation results in upregulation of FOXP3 protein expression in murine CD4+ T cells. A the highly conserved TSDR in the murine FOXP3 locus is shown and the positions of the CpGs as well as the targeted regions for each sgRNA are indicated. **B** Treg-depleted CD4+ T cells were sorted by MACS from splenocytes of C57Bl/6 mice and transfected (or not, 'MOCK') with a pool of 12 dCas9-TET1CD plasmids, each carrying one sgRNA sequence, which together target the entire TSDR. On day 2 post transfection, cells expressing the mCherry transfection reporter ('TET1') or not ('mCherry-') were sorted and recultured. On day 8, FOXP3+ and FOXP3- cells were sorted (frequency of each population is indicated in the FACS plot) and analyzed for TSDR methylation. The methylation level of each of the 10 analyzed CpGs (boxes) is indicated using the indicated color code. The mean methylation level for the entire TSDR is given in %.

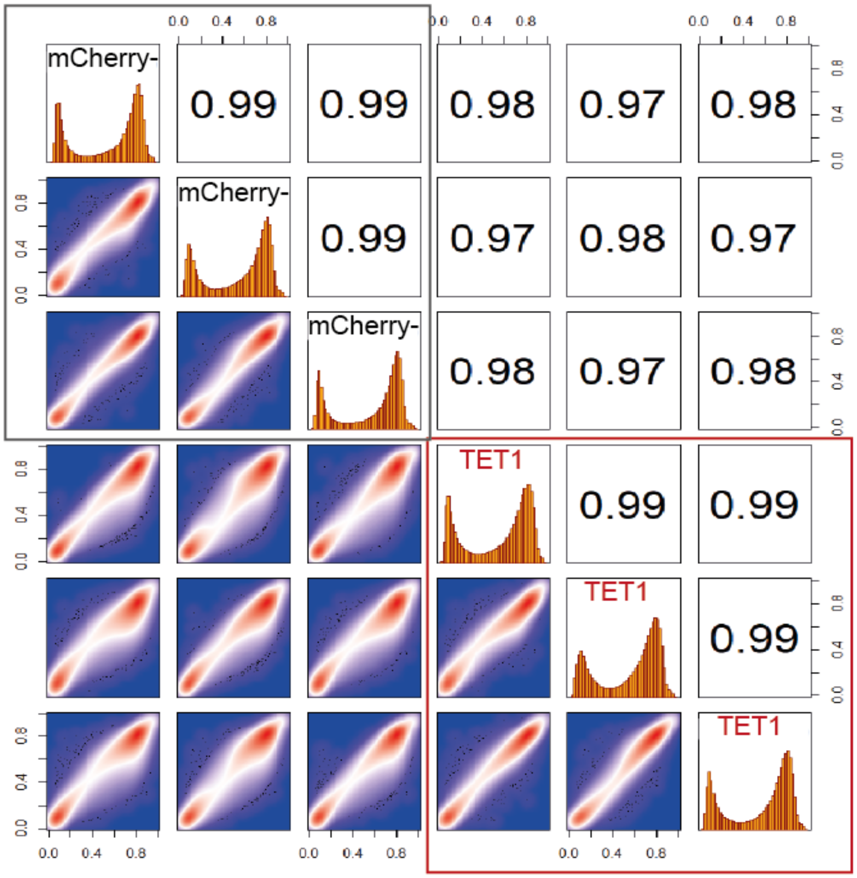

Supplementary Figure 3: Correlation Plots of genome-wide DNA methylation levels between three biological replicates of mCherry- and mCherry+ TET1 cells. Human naïve T cells were transfected with the pool of 12 dCas9-TET1CD plasmids and resorted according to mCherry expression on day 2 post transfection and recultured. Analysis for genome-wide DNA methylation levels was done on day 7 using the Illumina 850K EPIC bead-chip Array. Correlation plots are shown for each comparison. The r^2 values are indicated. The orange histograms indicate the distribution of CpG methylation levels in each sample.

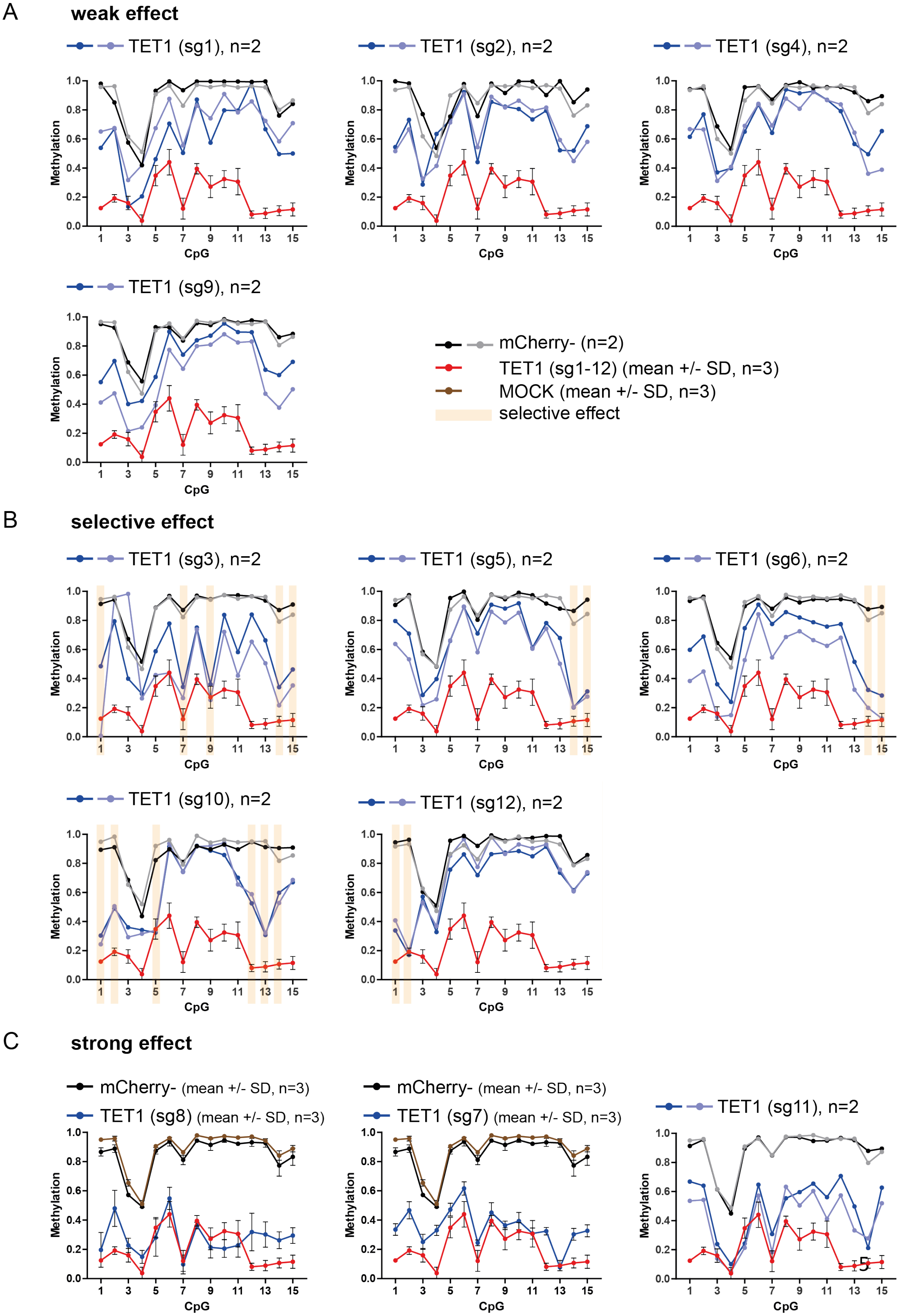
A
weak effect
TET1 (sg1), n=2
TET1 (sg2), n=2
TET1 (sg4), n=2
TET1 (sg9), n=2
mCherry- (n=2)
TET1 (sg1-12) (mean +/- SD, n=3)
MOCK (mean +/- SD, n=3)
selective effect
B
selective effect
TET1 (sg3), n=2
TET1 (sg5), n=2
TET1 (sg6), n=2
TET1 (sg10), n=2
TET1 (sg12), n=2
C
strong effect
mCherry- (mean +/- SD, n=3)
TET1 (sg8) (mean +/- SD, n=3)
mCherry- (mean +/- SD, n=3)
TET1 (sg7) (mean +/- SD, n=3)
TET1 (sg11), n=2
Methylation
CpG

Supplementary Figure 4: Transfection of dCas9-TET1CD guided by a single sgRNA result in various TSDR methylation patterns and can lead to complete TSDR de-methylation. Jurkat cells were analyzed on day 7 post transfection with the indicated sgRNA-guided dCas9-TET1CD complex and compared to indicated negative control samples. The plots are sorted according to the observed effects on TSDR de-methylation: **A** weak effects, **B** selective effects on selected TSDR CpGs (indicated by the orange box) and **C** strong effects on the entire TSDR.

Supplementary Table 1: Plasmids and single-guide (sg)RNA Sequences.

Addgene ID	Plasmid Name	sgRNA Sequence (5' - 3')
129027	pSp**dCas9-huTET1CD**-T2A-mCherry(PX458)	empty
129028	pSp**dCas9-hudTET1CD**-T2A-mCherry(PX458)	empty
129029	pSp**dCas9-huTET1CD**-T2A-mCherry(PX458)-**SghuTSDR-1**	GTCTGTGGTTTTGAGATTCT
129030	pSp**dCas9-huTET1CD**-T2A-mCherry(PX458)-**SghuTSDR-2**	TTATACTATTTGAAGACCCC
129031	pSp**dCas9-huTET1CD**-T2A-mCherry(PX458)-**SghuTSDR-3**	GGGCCAGGTCTTCAGAGCTA
129032	pSp**dCas9-huTET1CD**-T2A-mCherry(PX458)-**SghuTSDR-4**	TGTGGTAGGTGATGTCCATC
129033	pSp**dCas9-huTET1CD**-T2A-mCherry(PX458)-**SghuTSDR-5**	TGGGTGCTGGTGGATGTGGT
129034	pSp**dCas9-huTET1CD**-T2A-mCherry(PX458)-**SghuTSDR-6**	GCAGCAGGCTTGGCCCAGGT
129035	pSp**dCas9-huTET1CD**-T2A-mCherry(PX458)-**SghuTSDR-7**	GTTCTCGGAACGAAACCTGT
129036	pSp**dCas9-huTET1CD**-T2A-mCherry(PX458)-**SghuTSDR-8**	GGGCTTCATCGACACCACGG
129037	pSp**dCas9-huTET1CD**-T2A-mCherry(PX458)-**SghuTSDR-9**	TCATGGCGGCCGGATGCGCC
129038	pSp**dCas9-huTET1CD**-T2A-mCherry(PX458)-**SghuTSDR-10**	TTCCGCCATTGACGTCATGG
129039	pSp**dCas9-huTET1CD**-T2A-mCherry(PX458)-**SghuTSDR-11**	AGTCGGGGGCTGTGACAACA
129040	pSp**dCas9-huTET1CD**-T2A-mCherry(PX458)-**SghuTSDR-12**	TCTGAGAAACCCAGTCAGAA
129041	pSp**dCas9-hudTET1CD**-T2A-mCherry(PX458)-**SghuTSDR-1**	GTCTGTGGTTTTGAGATTCT
129042	pSp**dCas9-hudTET1CD**-T2A-mCherry(PX458)-**SghuTSDR-2**	TTATACTATTTGAAGACCCC
129043	pSp**dCas9-hudTET1CD**-T2A-mCherry(PX458)-**SghuTSDR-3**	GGGCCAGGTCTTCAGAGCTA
129044	pSp**dCas9-hudTET1CD**-T2A-mCherry(PX458)-**SghuTSDR-4**	TGTGGTAGGTGATGTCCATC
129045	pSp**dCas9-hudTET1CD**-T2A-mCherry(PX458)-**SghuTSDR-5**	TGGGTGCTGGTGGATGTGGT
129046	pSp**dCas9-hudTET1CD**-T2A-mCherry(PX458)-**SghuTSDR-6**	GCAGCAGGCTTGGCCCAGGT
129047	pSp**dCas9-hudTET1CD**-T2A-mCherry(PX458)-**SghuTSDR-7**	GTTCTCGGAACGAAACCTGT
129048	pSp**dCas9-hudTET1CD**-T2A-mCherry(PX458)-**SghuTSDR-8**	GGGCTTCATCGACACCACGG
129049	pSp**dCas9-hudTET1CD**-T2A-mCherry(PX458)-**SghuTSDR-9**	TCATGGCGGCCGGATGCGCC
129050	pSp**dCas9-hudTET1CD**-T2A-mCherry(PX458)-**SghuTSDR-10**	TTCCGCCATTGACGTCATGG
129051	pSp**dCas9-hudTET1CD**-T2A-mCherry(PX458)-**SghuTSDR-11**	AGTCGGGGGCTGTGACAACA
129052	pSp**dCas9-hudTET1CD**-T2A-mCherry(PX458)-**SghuTSDR-12**	TCTGAGAAACCCAGTCAGAA
129053	pSp**dCas9-huTET1CD**-T2A-mCherry(PX458)-**SgmouseTSDR-1**	CTTGAGATTCTAAAATCCGT
129054	pSp**dCas9-huTET1CD**-T2A-mCherry(PX458)-**SgmouseTSDR-2**	CTACAGCATTTGAAGACTCA
129055	pSp**dCas9-huTET1CD**-T2A-mCherry(PX458)-**SgmouseTSDR-3**	AGACAGAATCGATAGAACTT
129056	pSp**dCas9-huTET1CD**-T2A-mCherry(PX458)-**SgmouseTSDR-4**	TGTGGTAGGTGACGTCCATC
129057	pSp**dCas9-huTET1CD**-T2A-mCherry(PX458)-**SgmouseTSDR-5**	TGGGTGCTAGCGGATGTGGT
129058	pSp**dCas9-huTET1CD**-T2A-mCherry(PX458)-**SgmouseTSDR-6**	GTAGCCGGATAGGCCCAGGT
129059	pSp**dCas9-huTET1CD**-T2A-mCherry(PX458)-**SgmouseTSDR-7**	CTTCTCGGAACGAAACCTGT
129060	pSp**dCas9-huTET1CD**-T2A-mCherry(PX458)-**SgmouseTSDR-8**	GGGCTTCATCGGCAACAAGG
129061	pSp**dCas9-huTET1CD**-T2A-mCherry(PX458)-**SgmouseTSDR-9**	TCATGGCGGCCGGATGCATT
129062	pSp**dCas9-huTET1CD**-T2A-mCherry(PX458)-**SgmouseTSDR-10**	TTCTGCCATTGACGTCATGG
129063	pSp**dCas9-huTET1CD**-T2A-mCherry(PX458)-**SgmouseTSDR-11**	GCCCAGATGTAGACCCCGAT
129064	pSp**dCas9-huTET1CD**-T2A-mCherry(PX458)-**SgmouseTSDR-12**	CTTCTAAGAAACAGTCAAAC

Supplementary Table 2: Antibodies used in the study.

Antigen	Clone	Conjugate	Purpose	Supplier
CD3 (human)	UCHT1	---	stimulation (plate-coating)	BD Biosciences
CD28 (human)	CD28.2	---	stimulation (plate-coating)	BD Biosciences
CD3 (human)	UCHT1	AF700	staining	BioLegend
CD4 (human)	OKT4	BV510	staining	BioLegend
CD25 (human)	M-A251	PE	staining	BioLegend
CD127 (human)	HIL-7R-M21	APC	staining	BioLegend
CD45RO (human)	UCHL1	Pacific Blue	staining	BioLegend
CD45RA (human)	2H4	PE/Cy7	staining	Beckman Coulter
CXCR3 (human)	G025H7	AF488	staining	BioLegend
FOXP3 (human)	259D	Pacific Blue	staining	BioLegend
CTLA-4 (human)	BNI3	APC	staining	Miltenyi Biotec
CD137 (human)	4B4-1	VioBright FITC	staining	Miltenyi Biotec
CD154 (human)	5C8	APC	staining	Miltenyi Biotec
CD3ε (mouse)	145-2C11	---	stimulation (plate-coating)	DRFZ
CD28 (mouse)	37,51	---	stimulation (plate-coating)	DRFZ
CD25 (mouse)	PC61	APC	staining	BD Biosciences
Foxp3 (mouse)	FJK-16s	eFluor 450	staining	Thermo Fisher Scientific
Interferon-γ	4S.B3	BV605	staining	BioLegend
CD40	clone: HB14	---	surface stabilization	Miltenyi Biotec
CD28	15E8	---	stimulation (soluble)	Miltenyi Biotec
